# Triptolide represses oral cancer cell proliferation, invasion, migration, and angiogenesis in co-inoculation with U937 cells

**DOI:** 10.1007/s00784-016-1808-1

**Published:** 2016-04-13

**Authors:** Cheng-Yu Yang, Chih-Kung Lin, Gu-Jiun Lin, Cheng-Chih Hsieh, Shing-Hwa Huang, Kuo-Hsing Ma, Yi-Shing Shieh, Huey-Kang Sytwu, Yuan-Wu Chen

**Affiliations:** 1 Graduate Institute of Life Sciences, National Defense Medical Center, Taipei, Taiwan; 2 Division of Anatomic Pathology, Taipei Tzu Chi Hospital, Taipei, Taiwan; 3 Department of Biology and Anatomy, National Defense Medical Center, Taipei, Taiwan; 4 Department of Pharmacy Practice, Tri-Service General Hospital, Taipei, Taiwan; 5 School of Dentistry, National Defense Medical Center, Taipei, Taiwan; 6 Graduate Institute of Microbiology and Immunology, National Defense Medical Center, Taipei, Taiwan; 7 Department of Oral and Maxillofacial Surgery, Tri-Service General Hospital, Taipei, Taiwan; 8 Graduate Institute of Medical Sciences, National Defense Medical Center, Taiwan, No. 161, Section 6, Min-Chuan East Road, Neihu 114, Taipei 114, Taiwan, People’s Republic of China

**Keywords:** Triptolide, Chemoprevention, Co-inoculate, U937 cell, Oral cancer cell

## Abstract

**Objectives:**

Advanced oral cancer is a major public health concern because of a lack of effective prevention and treatment. Triptolide (TPL), a diterpenoid triepoxide derived from the Chinese herb *Tripterygium wilfordii*, has been demonstrated to possess strong anticancer properties. In this study, we investigated whether TPL exerts anticancer effects on the tumor microenvironment of head and neck squamous cell carcinoma (HNSCC).

**Materials and methods:**

Human macrophage-like U937 cells were co-inoculated with oral cancer SAS cells in a noncontact transwell coculture system. Cytokine expression was detected using ELISA, and cell proliferation was detected using methylene blue. RNA levels were detected using qPCR. Protein levels were detected using Western blot analysis. In vivo experiments involved using xenografted NOD/SCID mice.

**Results:**

Our results demonstrated that TPL inhibited the growth of SAS cells co-inoculated with U937 cells in vitro and in vivo. TPL inhibited the invasion, migration ability, and angiogenesis of SAS cells co-inoculated with U937 cells. Expression of cytokines IL-6, IL-8, and TNF-α was induced by co-inoculation, but TPL repressed their expression.

**Conclusion:**

TPL suppressed the expression of cytokines IL-6, IL-8, and TNF-α, as well as tumor growth, invasion, migration, and angiogenesis in the co-inoculation of human tongue cancer cells with macrophage-like U937 cells.

**Clinical relevance:**

TPL is a potential candidate among novel chemotherapeutic agents or adjuvants for modulating tumor-associated macrophages in a tumor microenvironment of HNSCC.

## Introduction

Oral squamous cell carcinoma (OSQCC), the most common of oral malignancies, is a type of head and neck squamous cell carcinoma (HNSCC), which is the sixth most prevalent malignancy worldwide and the third most common cancer in developing countries [1–5]. Concurrent chemoradiotherapy has exhibited efficacy for organ preservation in head and neck cancer, but has resulted in limited improvement in survival rates. Discovering potential therapeutic drugs for advanced oral cancer is thus paramount.

Macrophages are widely distributed in the body, being produced by the differentiation of monocytes in tissues. Macrophages participate in both innate and adaptive immunity of vertebrate animals. In cancer patients, macrophages are often observed to infiltrate the extracellular space around cancer cells and are designated as tumor-associated macrophages (TAMs) [6, 7]. A study revealed that the increase in TAMs in patients suffering from head and neck cancer is associated with an increase in histopathological grades and tumor angiogenesis [8].

**Fig. 1:**
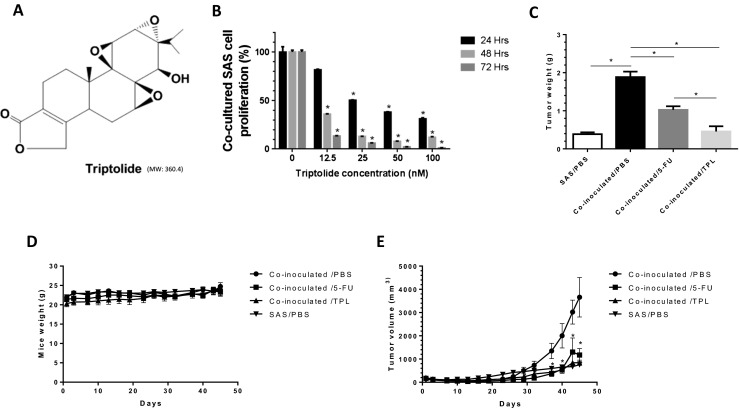
Triptolide represses oral cancer cell proliferation in co-inoculation with macrophage-like U937. **a** Chemical structure of Triptolide (TPL). **b** SAS co-inoculated with PMA-treated U937 cells were treated with indicated concentrations of TPL and then subjected to a methylene blue dye assay for 24, 48, and 72 h to analyze proliferation of cells. **c** In vivo, NOD/SCID mice bearing subcutaneous SAS co-inoculated with PMA-treated U937 cells were treated with PBS (*n* = 5), TPL (*n* = 5; 0.15 mg/kg/day), and 5-FU (*n* = 3; 6 mg/kg/day). Mice bearing SAS cells were treated with PBS as the control (*n* = 5). The average tumor weight of each group was compared with that of the control (*P < 0.05 by Student’s *t* test). **d** No significant change was observed in mice body weight compared with that of the control. **e** Diameters were measured twice a week for 45 days by using a Vernier caliper, and the tumor volume was calculated using the formula (L × W^2^)/2, where *W* is the shortest diameter and *L* is the longest diameter. Tumor volume compared with that of the control (*P < 0.05, data were analysis by one-way ANOVA). All data are expressed as mean±S.E.M

Triptolide (TPL, C_20_H_24_O_6_, Fig 1a), a diterpenoid triepoxide derived from the Chinese herb *Tripterygium wilfordii*, exerts effects against oral cancer [9]. Moreover, TPL exerts anticancer effects on multidrug-resistant KB-7D and KB-tax cells that overexpress multidrug resistance-associated protein (MRP), and the therapeutic effect of TPL can be enhanced by combining it with 5-fluorouracil (5-FU) [10]. Furthermore, we previously demonstrated that TPL represses HER2 and suppresses the downstream PI3K/Akt-signaling pathway [11]. Recently, we determined that TPL combined with ionizing radiation treatment exerted synergistic antitumor effects, particularly in vivo, and might be a promising combined therapy for advanced oral cancer [12].

In the present study, we demonstrated the chemopreventive therapeutic value of TPL. TPL suppressed the expression of cytokines IL-6, IL-8, and TNF-α and repressed tumor growth, invasion, migration, and angiogenesis in co-inoculation of human tongue cancer cells with macrophage-like U937 cells.

## Materials and methods

### Cells and chemicals

Human tongue squamous cell carcinoma cell line SAS was provided by Dr. Jeng-Fan Lo [13]. The cell line U937 (histiocytic lymphoma) was obtained from the Bioresource Collection and Research Center, Taiwan (derived from the American Type Culture Collection). U937 cells (1 × 10^5^ cells/mL) were differentiated to macrophage-like U937 cells by exposing them to 200 ng/mL of phorbol 12-myristate 13-acetate (PMA; Sigma, St. Louis, MO, USA). TPL (Calbiochem, San Diego, CA, USA; purity more than or equal to 95 % as determined using high-performance liquid chromatography) was dissolved in dimethyl sulfoxide as a 100-μM stock and added to cells at the indicated concentrations. 5-Fluorouracil (Sigma, F6627; purity more than or equal to 99 % as determined by high-performance liquid chromatography) was dissolved in phosphate buffer saline (PBS).

### Coculture in noncontact transwell system

Noncontact coculture transwell cell culture system was developed to study the cross biological activity of SAS and macrophage-like U937 cells. We modified the previously study methods [14]. In brief, the noncontact cocultured cells were prepared as follows: SAS cells were plated on the bottom of a six-well transwell cell culture system (pore size 0.4 μm) by using the complete media and culture environment as described. The U937 cells were cultured onto the membrane of transwell cell culture inserts and allowed to grow overnight by PMA treatment. On the next day, the cells were washed with media, and the macrophage-like U937 cells cultured on membrane transwell insert were placed into the six-well plate cultures containing the SAS to initiate the experiment.

### Growth inhibition assay

Cells in the logarithmic growth phase were cultured at a density of 1.5 × 10^5^ cells/well in a 24-well plate. The cells were exposed to various concentrations of TPL for 48 h. A methylene blue dye assay was used to evaluate the effect of TPL on cell growth, as described previously [9].

### Invasion assay

SAS and U937 cells (2.5 × 10^4^/chamber) were used for each invasion assy. The invasion assay was performed using modified Boyden chambers covered with a polycarbonate nucleopore membrane (Corning, Corning, NY, USA). Precoated filters (6.5 mm in diameter, 8-μm pores, 35 μg of Matrigel/chamber) were rehydrated, and 2.5 × 10^4^ cells in a medium were seeded into the upper part of each chamber. After 24 h of incubation, nonmigratory cells on the upper surface of the filter were wiped with a cotton swab, and migrated cells on the lower surface of the filter were fixed and stained with 0.125 % Commassie Blue in a methanol/acetic acid/water mixture (45:10:45, *v*/*v*/*v*). Random fields were counted under a light microscope.

### Wound healing assay

After co-inoculation of SAS cells with U937 cells, equal numbers of SAS cells were replated at high densities in six-well plates and grown overnight until reaching confluence. The next day, the monolayer was wounded using a 200-μL pipette tip. The media were changed to remove debris, and the wound was imaged at 0 h, and again after 4, 8, and 12 h. The average percentage of wound healing was determined according to three measurements of the wound area. For the wound healing assay, SAS cells were treated with 10 nM TPL for 4, 8, and 12 h prior to wounding.

### Vascular endothelial growth factor and cytokine analysis conducted using ELISA

SAS and U937 cells (1.5 × 10^5^) were grown in a complete RPMI medium (containing 10 % FBS) in triplicate for 2 days until they were 80–90 % confluent. After the cells were washed with an FBS-free medium, they were allowed to grow in a fresh RPMI medium containing 2 % FBS for 24 h. The medium was harvested and detected for human vascular endothelial growth factor (VEGF), IL-6, IL-8, and TNF-α by using ELISA kits (all eBioscience). The results were expressed as picogram per milliliter of the growth medium. The final concentrations of VEGF, IL-6, IL-8, and TNF-α were estimated by subtracting the obtained values from those of the control.

### Quantitative real-time PCR

Total RNA was extracted from the cells by using TRIzol (Life Technologies). Five micrograms of RNA from each sample were then reverse-transcribed using Superscript III Reverse Transcriptase (Life Technologies). Real-time quantitative PCR (RT-qPCR) was performed using SYBR Green PCR Master Mix (Life Technologies) and an ABI 7500 Fast detection system (Life Technologies). The RT-qPCR primers used were as follows: *MMP-9* forward, 5′-TCTTCCAGTACCGAGAGAAAG-3′; reverse, 5′- AGGATGTCATAGGTCACGTAG-3′; *E-cadherin* forward, 5′-ACA GCC CCG CCT TAT GAT T-3′; reverse, 5′-TCG GAA CCG CTT CCT TCA-3′; *vimentin* forward, 5′-AGTCCACTGAGTACCGGAGAC-3′; reverse, 5′-CATTTCACGCATCTGGCGTTC-3′); *snail* forward, 5′-CCC CAA TCG GAA GCC TAA CT-3′; reverse, 5′-GCT GGA AGG TAA ACT CTG GAT TAG A-3′; *VEGF* forward, 5′-GCTCTACCTCCACCATGCCA-3′; reverse, 5′-CACCACTTCGTGATGATTCTG-3′); *IL-6* forward, 5′-CCT TCC AAA GAT GGC TGA AA-3′; reverse, 5′-CAG GGG TGG TTA TTG CAT CT-3′); *IL-8* forward, 5′-ATG ACT TCC AAG CTG GCC GTG-3′; reverse, 5′-TCT CAG CCC TCT TCA AAA ACT-3′; *TNF-α* forward, 5′-AGG CGG TGC TTG TTC CTC A-3′; reverse, 5′-GTT CGA GAA GAT GAT CTG ACT GCC-3′; *GAPDH* forward, 5′-GGA AGG TGA AGG TCG GAG TCA-3′; reverse, 5′-GTC ATT GAT GGC AAC AAT ATC CAC T-3′.

### Protein extraction and Western blot analysis

The cells were lysed directly in an RIPA buffer (Millipore) supplemented with protease and phosphatase inhibitors (Sigma). The relative protein concentration was determined using a BCA protein assay kit (Thermo Scientific). For each lane of 8 to 10 % SDS–PAGE gel, 50 μg of cell lysate protein was loaded, separated, and transferred onto a polyvinyldifluoride (PVDF) membrane (Millipore). The membranes were then probed using specific antibodies against Matrix metallopeptidase 9 (MMP-9) (Abcam, ab38898), E-cadherin (BD Biosciences, 610,181), vimentin (Abcam, ab92547), snail protein (Cell Signaling, #3879), and β-actin (BioVision, 3598–100).

### Xenograft tumor model

Six-week-old NOD.CB17 Prkdcscid/J (National Laboratory Animal Center, Taiwan) mice were maintained in a microisolator in pathogen-free conditions. The mice were divided into four groups; each mouse in each group (*n* = 5; 5-FU, *n* = 3) was subcutaneously injected with 2 × 10^6^ of both SAS and SAS cocultured with macrophage-like U937 cells. Three days later, the mice in each group were further treated with TPL (0.15 mg/kg/day) and 5-FU (12 mg/kg/day), and a vehicle control (PBS) was then separately administered to the groups through intraperitoneal (ip) injection. The size of the transplanted tumors was measured using gauged calipers twice a week, and the tumor volume was calculated using the following formula: volume (*V*) = 1/2 × (length × width^2^). At the end of the treatment, the mice were sacrificed, and the tumors were removed, weighed, and photographed. The experiments were conducted in accordance with institutional guidelines and were approved by NDMC’s Institutional Animal Care and Use Committee (approval number: IACUC-14-048).

### Statistical analyses

All data were expressed as mean ± S.E.M. (standard error of the mean) of at least three determinations, unless otherwise stated. The differences between the two groups were determined using the Student’s *t* test or one-way ANOVA.

## Results

### Triptolide represses oral cancer cell proliferation in co-inoculation with macrophage-like U937 cells, both in vitro and in vivo

Tumor-associated macrophages induce the proliferation of cancer. We first tested whether TPL inhibited the growth of SAS cells co-inoculated with macrophage-like U937 cells. We then cocultured SAS cells with PMA-treated U937 cells in a noncontact system. After 24, 48, and 72 h, the growth was inhibited after treatment with various concentrations (0, 12.5, 25, 50, and 100 nM) of TPL, and the cell survival proportion was 100, 81.7, 50.3, 38.1, and 31.1 % at 24 h, respectively (Fig. 1b).

To further assess the therapeutic effect of TPL in vivo, we established a xenograft tumor model in which SAS oral cancer cells were co-inoculated with PMA-treated U937 cells. Tumor-bearing mice were randomly divided into four groups and treated with a vehicle (PBS) or TPL alone (0.15 mg/kg/day); 5-FU was used as the positive control (Fig. 1c). SAS co-inoculated with PMA-treated U937 cell xenografts treated with TPL were weighed (0.46 ± 0.28 g) and compared with the control group (1.88 ± 0.21 g) (*P* = 0.0005; <0.05 vs control). Xenografts from the TPL-treated group decreased 75.79 % in weight (Fig. 1c). The body weight of the mice was monitored at 3-day intervals throughout the experiment; the control group was 23.2 ± 2.2 g, and the treated group was 24 ± 0.9 g. No significant change in mouse body weight was observed compared with that of the control (Fig. 1d). However, dynamically measuring tumor volume revealed that TPL exerted an inhibitory effect on xenografts of SAS co-inoculated with U937 cells (Fig. 1e) (*P* = 0.0200, 0.0066, 0.0063, 0.0068, 0.0078, 0.0032; <0.05 vs untreated control). These results revealed that TPL was effective against SAS co-inoculated with macrophage-like U937 cells both in vitro and in vivo.

### Triptolide represses the invasive ability of oral cancer cells in co-inoculation with macrophage-like U937 cells

**Fig. 2:**
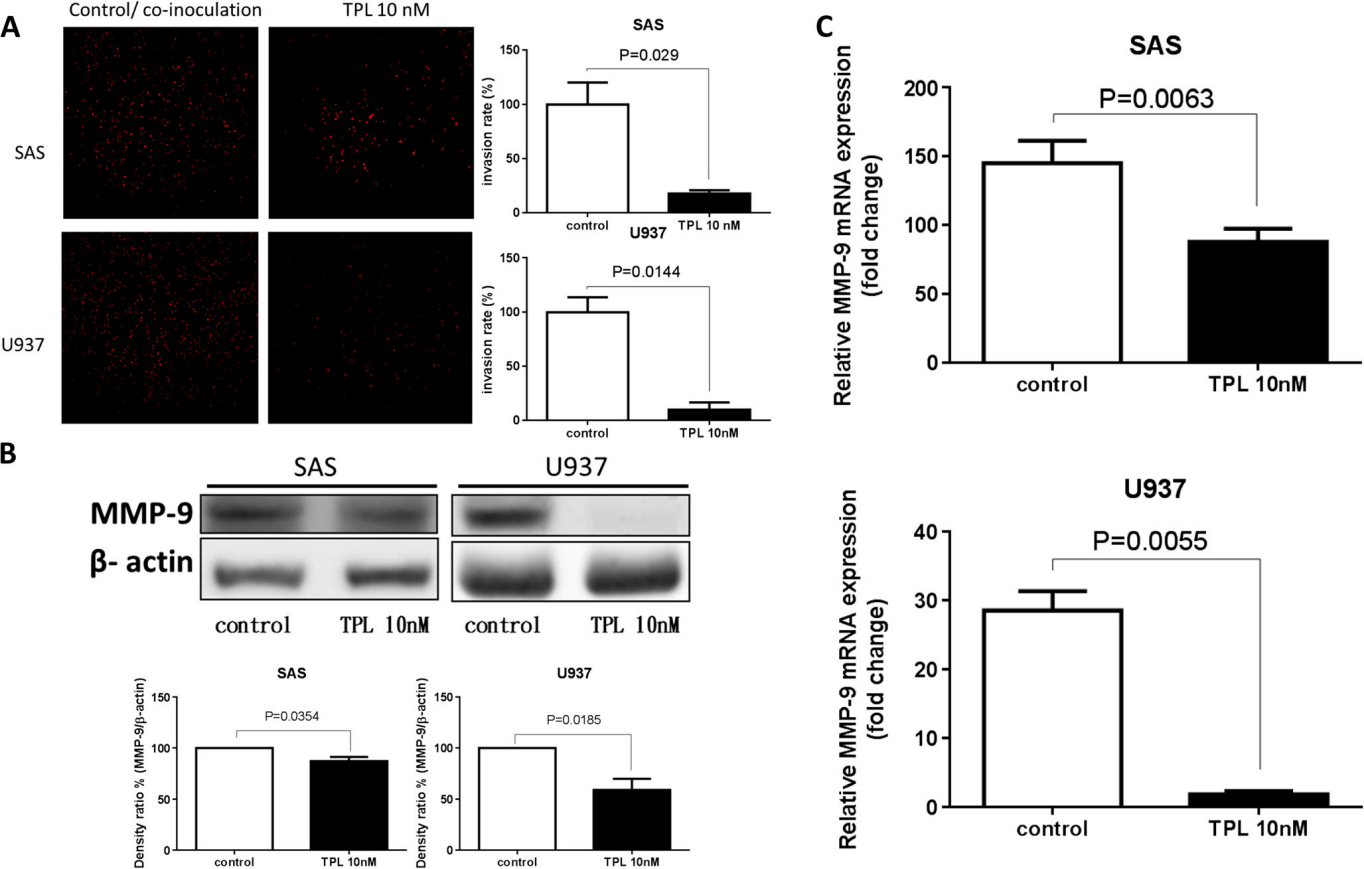
Triptolide represses oral cancer cell invasive ability in co-inoculation with macrophage-like U937. **a** Through the invasion assay, both SAS and PMA-treated U937 revealed a decreased invasive ability after TPL treatment compared with that of the control. **b** Effects of TPL on MMP-9 expression through a Western immunoblotting assay. **c** Effects of TPL on MMP-9 expression according to Q-PCR (*P* < 0.05 by Student’s *t* test)

For the invasion assay, the upper parts of the transwells were coated with Matrigel. Adding TPL resulted in a reduction of approximately 80 % for the penetration of both SAS and PMA-treated U937 cells through a Matrigel-coated membrane compared with that of the control group (Fig. 2a). Treatment with 0 and 10 nM TPL resulted in an SAS cell count of 100 and 17.5 ± 2.12 %, respectively (*P* = 0.0290; < 0.05 vs untreated control), and a PMA-treated U937 cell count of 100 % and 10.05 ± 4.75 %, respectively (*P* = 0.0144; < 0.05 vs untreated control) (Fig. 2a). Western blot analysis revealed that MMP-9 was downregulated compared with that of the control group. In SAS cells treated with 0 and 10 nM TPL, MMP-9 protein expression levels were 100 and 87 ± 5.89 %, respectively (Fig. 2B; *P* = 0.0354; < 0.05 vs untreated control). In PMA-treated U937 cells treated with 0 and 10 nM TPL, MMP-9 protein expression levels were 100 and 59 ± 15.12 %, respectively (Fig. 2b; *P* = 0.0185; <0.05 vs untreated control). Total RNA was isolated, and RT-PCR analyses of MMP-9 were performed. GAPDH was used as an internal control for RT-PCR. In TPL-treated cells, MMP-9 expression exhibited a decrease of approximately 50 % compared with that of the control (Fig. 2c). In SAS cells treated with 0 and 10 nM TPL, MMP-9 expression levels changed 145.24- and 88.07-fold, respectively (Fig. 2c; *P* = 0.0063; <0.05 vs untreated control). In PMA-treated U937 cells treated with 0 and 10 nM TPL, MMP-9 expression levels changed 28.56- and 1.82-fold, respectively (Fig. 2c; *P* = 0.0055; <0.05 vs untreated control).

### Triptolide represses the migration ability of oral cancer cells in co-inoculation with macrophage-like U937 cells

**Fig. 3:**
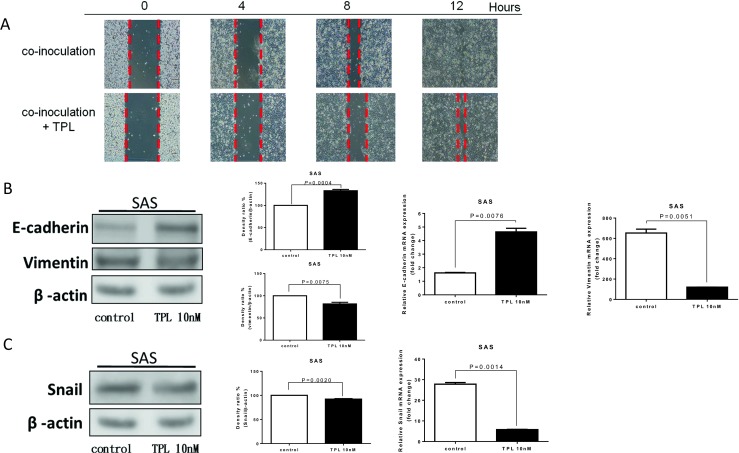
Triptolide represses oral cancer cell migration ability in co-inoculation with macrophage-like U937. **a** Through a wound healing assay, SAS cells demonstrated a decreased migration ability after TPL treatment compared with that of the control. Images of the wound were captured under ×100 magnifications by using a microscope. **b** Effects of TPL on E-cadherin and vimentin expression through a Western immunoblotting assay. After 10 nM TPL treatment for 48 h, E-cadherin increased and vimentin expression decreased compared with those of the control. **c** After 10 nM TPL treatment for 48 h, snail protein expression decreased compared with that of the control (*P* < 0.05 by Student’s *t* test)

For the wound healing assay, cells were incubated in a six-well plate and treated with TPL for 4 h. Images of the wound were captured under ×100 magnification by using a microscope. Cell migration was significantly decreased by TPL treatment compared with that of the control group, and the wound was imaged at 0 h and again after 4, 8, and 12 h (Fig. 3a). Western blot analysis revealed that E-cadherin was upregulated and vimentin was downregulated compared with those of the control group. In cells treated with 0 and 10 nM TPL, E-cadherin protein expression levels were 133 ± 4.32 and 100 %, respectively (*P* = 0.0004; <0.05 vs untreated control), whereas vimentin protein expression levels were 81.7 ± 5.19 and 100 %, respectively (Fig. 3b; *P* = 0.0075; <0.05 vs untreated control). GAPDH was used as an internal control for RT-PCR. Q-PCR revealed that compared with those of the control, E-cadherin exhibited 1.6- and 4.6-fold expression in cells treated with 0 and 10 nM TPL, respectively (*P* = 0.0076; <0.05 vs untreated control), whereas vimentin exhibited 653- and 120-fold expression in cells treated with 0 and 10 nM TPL, respectively (*P* = 0.0051; <0.05 vs untreated control) (Fig. 3b). Snail protein was downregulated compared with that of the control group. In cells treated with 0 and 10 nM TPL, the expression levels of snail protein were 100 and 92.1 ± 1.54 %, respectively (Fig. 3c; *P* = 0.0020; <0.05 vs untreated control). Q-PCR revealed that compared with that of the control, snail protein exhibited 27.8- and 5.8-fold expression in the cells treated with 0 and 10 nM TPL, respectively (Fig. 3c; *P* = 0.0014; < 0.05 vs untreated control).

### Triptolide represses the angiogenesis ability of oral cancer cells in co-inoculation with macrophage-like U937 cells

**Fig. 4:**
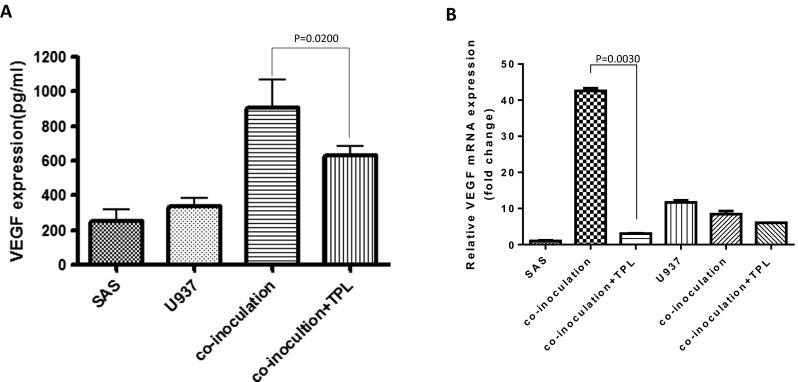
Triptolide represses oral cancer cell angiogenesis ability in co-inoculation with macrophage-like U937. **a** Effects of TPL on VEGF expression by ELISA. After 10 nM TPL treatment for 48 h, VEGF protein expression decreased compared with that of the control. **b** Effects of TPL on VEGF expression by Q-PCR. The data revealed the VEGF is a major expression from SAS and downregulated by TPL treatment compared with that of the control (*P* < 0.05 by Student’s *t* test)

In co-inoculation with PMA-treated U937 cells, VEGF was downregulated in the TPL-treated group compared with that of the control group (co-inoculation U937 cells). In cells treated with 0 and 10 nM TPL, VEGF exhibited expression protein levels of 100 and 74 ± 8.48 %, respectively (Fig. 4a). Total RNA was isolated, and RT-PCR analyses of VEGF were performed. GAPDH was used as an internal control for RT-PCR. We determined that VEGF was predominantly secreted by SAS cells (not by PMA-treated U937 cells) in the co-inoculation of both cell lines. According to the Q-PCR results, TPL-treatment resulted in a *VEGF* reduction of approximately 90 % compared with that of the control (SAS co-inoculation) (Fig. 4b).

### Triptolide represses cytokine expression in co-inoculation of SAS cells with macrophage-like U937 cells

**Fig. 5:**
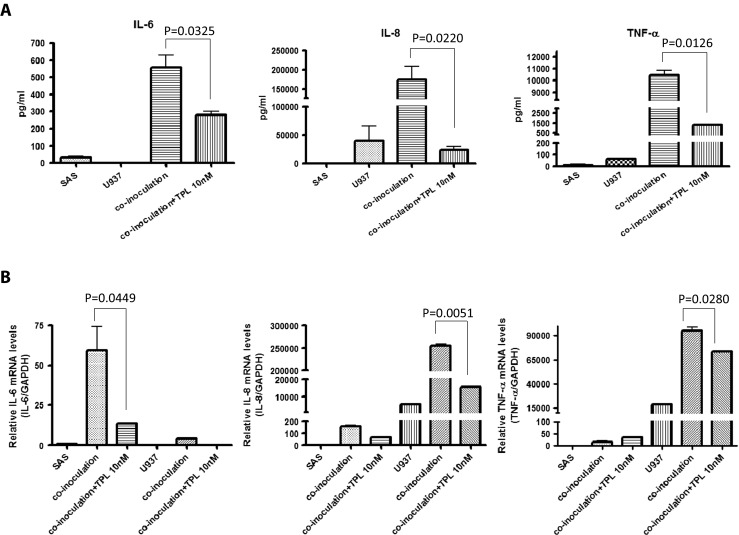
Triptolide represses these cytokines expression in SAS co-inoculation with macrophage-like U937. **a** The cytokine expression of IL-6, IL-8, and TNF-α were induced by co-inoculation, and the TPL (10 nM) repressed these cytokines expression by ELISA. **b** IL-6 was predominantly secreted from SAS cells; IL-8 and TNF-α were predominantly secreted from U937, detected using Q-PCR. All these cytokines were repressed by TPL compared with those of the control (*P* < 0.05 by Student’s *t* test)

Cytokines IL-6, IL-8, and TNF-α were abundantly secreted in the co-inoculation of SAS cells with PMA-treated U937 cells, but TPL repressed these cytokines according to the results of the ELISA (Fig. 5a). In cells treated with 0 and 10 nM TPL, IL-6 concentrations were 557 and 282 pg/mL, respectively; IL-8 concentrations were 175 473 and 24 495 pg/mL, respectively; and TNF-α concentrations were 10 440 and 1316 pg/mL, respectively. According to the Q-PCR results, IL-6 was predominantly secreted by SAS cells, whereas IL-8 and TNF-α were predominantly secreted by PMA-treated U937 cells (Fig. 5b). All these cytokines were repressed by TPL in contrast to the control. The Q-PCR results revealed that in cells treated with 0 and 10 nM TPL, IL-6 exhibited 60- and 10-fold expression, respectively, IL-8 exhibited 250 000- and 13 000-fold expression, respectively, and TNF-α exhibited 90 000- and 70 000-fold expression, respectively, compared with those of the control (SAS) (Fig. 5b).

## Discussion

Our previous study revealed that TPL can inhibit tumor growth by inducing apoptosis in cancer cells [9]. In the present study, we demonstrated that TPL can inhibit SAS cell growth in co-inoculated with macrophage-like U937 cells, in vitro and in vivo (Fig. 1). A previous study demonstrated that PC-3 prostate cancer cells and U937 promonocytic cells enhanced tumor growth and increased tumor angiogenesis [15]; they determined that IL-4 was a crucial cytokine regulating the differentiation of monocytes and macrophages into TAMs in prostate cancer cells. Stimulating U937 cells with IL-4 resulted in an increase in tumor growth in vivo and stimulated angiogenesis within the tumor bed.

A study revealed that MMPs play a key role for tumor growth, progression, metastasis, and angiogenesis [16]. Both MMP-2 and MMP-9 are mainly involved in tumor metastasis. In human fibrosarcoma cells (HT-1080), TPL can modulate the expression and activity of both MMP-2 and MMP-9, and it can reduce invasiveness by directly lowering MMP-9 gene expression and activity [17]. Here, we demonstrated that TPL inhibited not only the invasive ability of SAS cells but also the invasive ability of macrophage-like U937 cells in co-inoculation (Fig. 2a). Both protein (Fig. 2b) and gene (Fig. 2c) expressions were downregulated by TPL (10 nM) treatment for 24 and 48 h.

In ovarian cancer cells SKOV3 and A2780, TPL (15 nM) inhibited cell migration but enhanced E-cadherin expression in tumors in a dose-dependent manner [18]. In the present study, we demonstrated that TPL inhibited the migratory ability of SAS cells in co-inoculation with macrophage-like U937 cells (Fig. 3a). E-cadherin was upregulated and vimentin was downregulated after TPL treatment of SAS cells (Fig. 3b). Snail protein was also downregulated after TPL treatment of SAS cells (Fig. 3c). These epithelium-mesenchymal transition-related proteins were modulated by TPL treatment and exhibited consistent tumorigenicity.

VEGF is a signal protein produced by cells that stimulates angiogenesis. In pancreatic cancer cells (PANC-1), TPL decreased the expression of VEGF both in vitro and in vivo in a time- and concentration-dependent manner [19]. We determined that VEGF increased substantially in a culture medium, and TPL decreased VEGF levels in co-inoculation with macrophage-like U937 cells (Fig. 4a). We also demonstrated that VEGF was predominantly secreted by SAS cells (not by macrophage-like U937 cells), according to the Q-PCR results (Fig. 4b).

Cancer cells produce several cytokines that act on the surrounding interstitial cells, which can build a microenvironment for the growth and metastasis of cancer cells. We determined that cytokines IL-6, IL-8, and TNF-α were abundantly secreted in the co-inoculation of SAS cells with macrophage-like U937 cells, as detected using an ELISA (Fig. 5a). The Q-PCR results demonstrate that IL-6 was predominantly secreted by SAS cells (not by macrophage-like U937 cells) (Fig. 5b). IL-6 is a cytokine secreted by T cells and macrophages to stimulate immune response, but elevated expression of IL-6 has been detected in multiple tumors [20]. Many studies have reported a high concentration of IL-6 in the serum of patients with head and neck cancer and have associated high IL-6 levels with a poor prognosis [21, 22]. These results have suggested that IL-6 is likely to invade and metastasize, as well as promote immune unresponsiveness (Figs. 2 and 3).

The Q-PCR results also demonstrate that IL-8 was predominantly secreted by macrophage-like U937 cells (not by SAS cells) (Fig. 5b). IL-8 is a neutrophil chemotactic factor produced by macrophages and epithelial cells; it induces chemotaxis and phagocytosis in target cells and is a potent promoter of angiogenesis. IL-8 protein typically increases in the serum of cancer patients and affects the proliferation, migration, angiogenesis, and metastasis of cancer cells [20, 23, 24] Local IL-8 production is related to malignancies and tumor progression; thus, elevated IL-8 levels in serum are indicative of a malignant process. High IL-8 levels are typically observed in high-grade peritumoral fluids rather than in low-grade tumors and benign conditions. Hence, IL-8 in peritumoral fluid must be considered when assessing tumor character and monitoring tumor progression or remission status.

In this study, TNF-α was predominantly secreted by macrophage-like U937 cells (not by SAS cells), according to the Q-PCR results (Fig. 5b). TNF-α is the most critical proinflammatory cytokine involved in cell growth, differentiation, and apoptosis [25, 26] and has been reported to play a critical role in carcinogenesis [26]. Consistent with these reports, numerous studies have indicated that chronic inflammation and proinflammatory mediators including TNF-α might increase the risk of malignancy [27].

TAMs have been implicated in promoting tumor growth and progression. Thus, macrophages are at the center of the invasion microenvironment and are a crucial drug target for cancer therapy [6]. Macrophages do not harbor malignant mutations and therefore exhibit a stable genome; thus, they are unlikely to develop drug resistance. This makes them an optimal target for cytostatic treatment of tumor progression to malignancy, using small molecule inhibitors of selected macrophage functions. Studying the signaling pathways that allow macrophages to contribute to tumor progression can lead to new insights into the evolution of the microenvironments supporting invasion and metastasis, thereby providing targets for anticancer therapies [6].

This study indicates the potential anticancer role of TPL, a compound that exhibited antitumor effects in co-inoculation of human tongue cancer cells with macrophage-like U937 cells. Thus, TPL is a potential candidate among novel chemotherapeutic agents or adjuvants for modulating TAMs in a tumor microenvironment.

## References

[1] Pentenero M, Gandolfo S, Carrozzo M (2005). Importance of tumor thickness and depth of invasion in nodal involvement and prognosis of oral squamous cell carcinoma: a review of the literature. Head Neck.

[2] Chen YJ (2004). Genome-wide profiling of oral squamous cell carcinoma. J Pathol.

[3] Daley T, Darling M (2003). Nonsquamous cell malignant tumours of the oral cavity: an overview. J can Dent Assoc.

[4] Lyons AJ, Jones J (2007). Cell adhesion molecules, the extracellular matrix and oral squamous carcinoma. Int J Oral Maxillofac Surg.

[5] Jemal A (2009). Cancer statistics 2009. Ca Cancer J Clin,.

[6] Condeelis J, Pollard JW (2006). Macrophages: obligate partners for tumor cell migration, invasion, and metastasis. Cell.

[7] Lewis CE, Pollard JW (2006). Distinct role of macrophages in different tumor microenvironments. Cancer Res.

[8] El-Rouby DH (2010). Association of macrophages with angiogenesis in oral verrucous and squamous cell carcinomas. J Oral Pathol Med.

[9] Chen YW (2009). Triptolide exerts anti-tumor effect on oral cancer and KB cells in vitro and in vivo. Oral Oncol.

[10] Herzog A (2013). PI3K/mTOR inhibitor PF-04691502 antitumor activity is enhanced with induction of wild-type TP53 in human xenograft and murine knockout models of head and neck cancer. Clin Cancer Res.

[11] Ou CC (2012). Triptolide transcriptionally represses HER2 in ovarian cancer cells by targeting NF-kappaB. Evid Based Complement Alternat Med.

[12] Chen YW (2014). Enhanced anti-tumor activity of triptolide in combination with irradiation for the treatment of oral cancer. Planta Med.

[13] Lo JF (2011). The epithelial-mesenchymal transition mediator S100A4 maintains cancer-initiating cells in head and neck cancers. Cancer Res.

[14] Kumar R (2012). Correction: co-culture of retinal and endothelial cells results in the modulation of genes critical to retinal neovascularization. Vasc Cell.

[15] Craig M, Ying C, Loberg RD (2008). Co-inoculation of prostate cancer cells with U937 enhances tumor growth and angiogenesis in vivo. J Cell Biochem.

[16] Gialeli C, Theocharis AD, Karamanos NK (2011). Roles of matrix metalloproteinases in cancer progression and their pharmacological targeting. Febs J.

[17] Yang S (2011). Inhibitive effect of triptolide on invasiveness of human fibrosarcoma cells by downregulating matrix metalloproteinase-9 expression. Asian Pac J Trop Med.

[18] Zhao H (2012). Triptolide inhibits ovarian cancer cell invasion by repression of matrix metalloproteinase 7 and 19 and upregulation of E-cadherin. Exp Mol Med.

[19] Ma JX (2013). Triptolide induces apoptosis and inhibits the growth and angiogenesis of human pancreatic cancer cells by downregulating COX-2 and VEGF. Oncol Res.

[20] Kishimoto T (2005). Interleukin-6: from basic science to medicine—40 years in immunology. Annu Rev Immunol.

[21] Chen Z (1999). Expression of proinflammatory and proangiogenic cytokines in patients with head and neck cancer. Clin Cancer Res.

[22] Duffy SA (2008). Interleukin-6 predicts recurrence and survival among head and neck cancer patients. Cancer.

[23] Kotyza J (2012). Interleukin-8 (CXCL8) in tumor associated non-vascular extracellular fluids: its diagnostic and prognostic values. A review. Int J Biol Markers.

[24] Desai S, Laskar S, Pandey BN (2013). Autocrine IL-8 and VEGF mediate epithelial-mesenchymal transition and invasiveness via p38/JNK-ATF-2 signalling in A549 lung cancer cells. Cell Signal.

[25] Waters JP, Pober JS, Bradley JR (2013). Tumour necrosis factor in infectious disease. J Pathol.

[26] Aggarwal BB, Gupta SC, Kim JH (2012). Historical perspectives on tumor necrosis factor and its superfamily: 25 years later, a golden journey. Blood.

[27] Li M, You Q, Wang X (2011). Association between polymorphism of the tumor necrosis factor alpha-308 gene promoter and colon cancer in the Chinese population. Genet Test Mol Biomarkers.

